# Celiac Disease as a Rare Cause of Membranous Nephropathy: A Case Report

**DOI:** 10.7759/cureus.13312

**Published:** 2021-02-12

**Authors:** Nicole Pestana, Carlota Vida, Pedro Vieira, José Durães, Gil Silva

**Affiliations:** 1 Nephrology Department, Hospital Central do Funchal, Funchal, PRT

**Keywords:** membranous nephropathy, celiac disease, nephrotic syndrome

## Abstract

Membranous nephropathy is the most common cause of nephrotic syndrome in adults. A non-negligible number of cases are associated with systemic conditions. We report a case of a 50-year-old man who presented with nephrotic syndrome six months after being diagnosed with celiac disease. Although the patient showed disappearance of circulating immunoglobulin A (IgA) anti-tissue transglutaminase antibodies following a gluten-free diet, he had a sudden onset of nephrotic syndrome presenting with severe hypoalbuminemia. Other secondary causes were promptly excluded leading to the assumption of celiac disease-associated membranous nephropathy with remission after treatment with angiotensin system blockade and a gluten-free diet. The goal of this case report is to alert the clinic towards this rare association aiming for an early diagnosis and adequate selection of long-term therapy.

## Introduction

Membranous nephropathy (MN) is the most common cause of nephrotic syndrome in white Caucasian adults, accounting for approximately 40% of cases [1]. MN is classically divided into two subgroups: primary and secondary MN. Secondary MN constitutes around 20% of all MN cases and occurs in the context of underlying conditions, namely malignancies, drugs, rheumatological diseases, and infections [2]. Celiac disease (CD) is one of the most common autoimmune disorders, with a reported prevalence of 0.5-1% of the general population [3]. Although the incidence of renal disease in CD patients is low and vice versa, this association has pathogenic aspects that can affect both conditions. Here we report a case of MN in a patient with CD and discuss the pitfalls of underlying investigation in this glomerular disease.

## Case presentation

**Figure 1:**
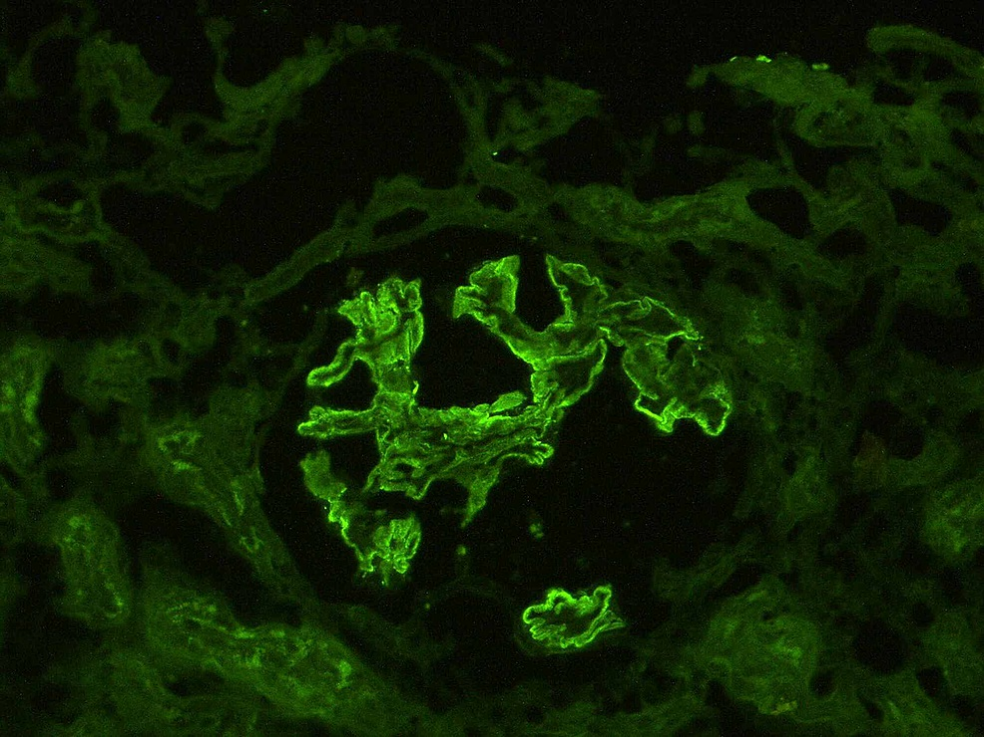
Immunoflurorescence microscopy Granular deposition of IgG along the outer surface of capillary walls

The authors present a case of a 50-year-old Caucasian man with CD diagnosed in January 2020 and heavy smoking habits (>25 cigarettes a day). He was referred to Nephrology Department in June 2020 due to recent onset of generalized edema with an 8 kg weight gain and deterioration of renal function. On physical exam he was normotensive and presented with bilateral pitting edema. Laboratory findings revealed decreased renal function (serum creatinine 1.5 mg/dL as opposed to six months earlier 1 mg/dL), severe hypoalbuminemia (albumin 17 g/L) and hypertriglyceridemia (triglycerides 550 mg/dL). Urinary analysis showed proteinuria (4+) with a 24h urine protein excretion of 30 g. Kidney ultrasound was unremarkable with normal kidneys. Considering a full-blown nephrotic syndrome with massive proteinuria, a renal biopsy was performed revealing a membranous nephropathy (Figure 1).

Given his known CD and possible relationship with glomerulonephritis, analytical investigation was extended and revealed disappearance of circulating immunoglobulin A (IgA) anti-tissue transglutaminase antibodies following a six-month gluten-free diet. At this point, the hypothetical diagnosis of secondary MN due to CD was temporarily postponed to exclude other further MN causes. Additional analysis revealed a low titer of antinuclear antibodies (ANA; with a non-specific nucleolar pattern) (titer 1:320) and negative anti-double-stranded DNA, rheumatoid factor, complement, anti-neutrophil cytoplasmic, and anti-phospholipase A2 receptor antibodies (anti-PLA2R). Supplementary studies to characterize the ANA pattern did not reveal any specific disease-associated autoantibodies. Nailfold capillaroscopy was performed, excluding Raynaud’s phenomenon. Hepatitis B, C, and HIV were also excluded. Thoracic and abdominal computed tomography scan, colonoscopy, and endoscopy were negative. Regarding his past medical history, the patient had never received gold salts or nonsteroidal anti-inflammatory drugs. Considering the exclusion of other secondary causes, the assumption of MN secondary to CD was made. A conservative approach was initiated by ramipril until 15 mg a day in addition to a gluten-free diet. Rituximab was considered but considering partial remission in two months to proteinuria under 6 g/day, the approach remained conservative. Although the low evidence of MN and CD association reported, the presentation of full blow nephrotic syndrome following CD diagnosis and the response to a gluten-free diet supported the diagnosis of CD-associated MN.

## Discussion

The association between CD and MN is rarely reported in the literature [4]. As both are immune-mediated diseases, they possibly share an autoimmune pathogenesis with simultaneous digestive and renal involvement. Some described an increase in the number of cases of chronic glomerulonephritis and renal insufficiency in patients with CD and this risk is greater in adults [5]. By far the main association between CD and glomerular disease is IgA nephropathy (IgAN) with 22-77% of patients with IgAN having IgA anti-gliadin antibodies and 3-4% of these having CD [6,7]. In our case report the patient had histological evidence of MN but no IgA deposits, excluding any resemblance to IgAN. In our opinion, the link between CD and MN is not unexpected. Although there was a different temporal onset of both diseases the patient had remission of massive proteinuria followed by disappearance of circulating IgA anti-tissue transglutaminase antibodies. In fact, there was remission of both diseases, as is seen in the majority of cases of secondary MN that remit when the original cause is addressed [8]. Anti-PLA2R autoantibodies are highly specific for primary MN and are found in approximately 75% of patients at time of diagnosis [9]. We would like to emphasize that our patient had negative anti-PLA2R leading us to secondary investigation as recommended by the 2012 Kidney Disease Improving Global Outcomes (KDIGO) guidelines [10]. ANAs have also been found in autoimmune diseases and cancer [11,12]. Its presence although unspecific alerts us to the possibility of systemic lupus erythematosus (SLE) which has a known association with CD [13], and systemic sclerosis (SS). The absence of clinical evidence of SLE (arthralgias, cutaneous lesions, photosensitivity, telangiectasia, hematological affection, or active urinary sediment) associated with the negative anti-DNA antibodies excludes this diagnosis. In addition, the absence of Raynaud’s phenomenon, skin changes, and unexplained dry month and eyes associated with negative specific antibodies disease do not contribute to the diagnosis of SS nor Sjogren syndrome. Considering this, concerns of neoplasms were raised. Cancer diagnosis accounts for 10% of secondary cases of MN, with lung and gastrointestinal systems the most frequent [14]. These are sometimes diagnosed in a non-specific nucleolar ANA pattern without identified autoantibody [12]. Regarding the patient's heavy smoking habits, MN-associated neoplasia was promptly studied and excluded. We concluded our investigation with exclusion of specific MN-implicated drugs (gold salt drugs and anti-nonsteroidal anti-inflammatory drugs). The assumption of secondary MN associated with CD was made with the patient entering partial remission after two months of increasing angiotensin blocker therapy and continuous gluten-free diet. Literature supports our case report in which the absence of anti-PLA2R is associated with a higher likelihood of clinical remission [15].

## Conclusions

Clinicians should be aware of the rare association between CD and MN. This must be considered in a patient presenting with improvement of nephrotic syndrome after angiotensin blockage therapy and gluten-free diet, despite the absence of IgA anti-tissue transglutaminase antibodies. Further studies are necessary to elucidate the possible common pathogenic mechanisms of both diseases.
